# Accuracy of transthoracic echocardiography in diagnosis of cardiac myxoma: single center experience

**DOI:** 10.2478/raon-2025-0007

**Published:** 2025-01-22

**Authors:** Polona Kacar, Nejc Pavsic, Mojca Bervar, Zvezdana Dolenc Strazar, Katja Prokselj

**Affiliations:** 1 Department of Cardiology, University Medical Center Ljubljana, Ljubljana, Slovenia; 2 Faculty of Medicine, University of Ljubljana, Ljubljana, Slovenia

**Keywords:** cardiac mass, cardiac myxoma, cardiovascular imaging, echocardiography

## Abstract

**Background:**

The differential diagnosis of cardiac myxomas (CM), the most common benign primary cardiac tumors, is broad and a thorough diagnostic workup is required to establish accurate diagnosis prior to surgical resection. Transthoracic echocardiography (TTE) is usually the first imaging modality used for diagnosis of suspected CM. In a single tertiary centre study, we sought to determine the accuracy, sensitivity, and specificity of TTE in the diagnosis of CM and to determine echocardiographic characteristics indicative of CM.

**Patients and methods:**

We retrospectively analyzed clinical, echocardiographic, and pathohistological findings of 73 patients consecutively admitted for suspected CM.

**Results:**

After diagnostic workup, 53 (73%) patients were treated surgically at our institution. Based on preoperative TTE, patients were divided into a CM group (n=45, 85%) and non-myxoma (NM) group. Of the 53 pathohistological specimens obtained during surgery, 39 (73%) were CM. The sensitivity and specificity of preoperative echocardiography were 97% and 50%, respectively. The overall accuracy was 85%. All NM tumors were found in an atypical location and 72% of CM were found in a typical position in the left atrium (p < 0.001). Tumors in NM group were significantly smaller than CM (24.3 ± 13.2 mm *vs*. 37.9 ± 18.3 mm, p = 0.017).

**Conclusions:**

Our study confirms very good accuracy of TTE in the diagnosis of CM. The most important echocardiographic characteristics to differentiate between CM and tumors of different etiology are tumor location and size. Smaller tumors presenting at an atypical location are less likely to be diagnosed as CM, and these require additional imaging modalities for accurate diagnosis.

## Introduction

Although rare, cardiac myxoma (CM) represents the most common benign primary cardiac tumor.^1^ Many patients are asymptomatic and CM is often an incidental finding.^2^ Potentially life-threatening complications such as tumor obstruction or embolization can occur, making accurate diagnosis crucial.^3,4^ However, diagnosis is challenging due to the broad differential diagnosis of CM, which includes other cardiac tumors and cardiac masses such as thrombi, vegetations, calcific lesions, and other rare conditions.

Transthoracic echocardiography (TTE) nowadays represents the most commonly used initial imaging modality in the diagnostic workup of CM. It provides information on tumor size, location, attachment point, morphology, mobility, and its relation to surrounding structures. The majority of CM are located in the left atrium, attached to the atrial septum in the region of the fossa ovalis. These are considered as typical CM, but atypical localizations outside the left atrium have been described in around 30%.^5^ Size and appearance (solid and round or polypoid) may also vary considerably in CM.^6^

TTE has an excellent detection rate for CM and a sensitivity of 90–96% in diagnosing CM has been reported.^4^ However, the heterogeneous morphological presentation leads to overlap with other cardiac masses and may affect the specificity and accuracy of TTE in CM diagnosis. Furthermore, TTE lacks tissue characterization.^7^ Multimodality cardiac imaging ensures a more detailed analysis. Ultimately, the final diagnosis is made by histopathological examination of the excised tumor.^8^

The aim of our single-center study was to evaluate the utility and accuracy of TTE in the diagnosis of CM and to determine echocardiographic characteristics indicative of pathohistologically confirmed CM.

## Patients and methods

The study was conducted in accordance with the Declaration of Helsinki (as revised in 2013). The study was approved by national ethics committee of Slovenia (NO.: 0120-512/2020-3) and informed consent was obtained from individual participants.

We retrospectively analyzed clinical, echocardiographic, and pathohistological findings of all consecutive adult patients (≥ 18 years of age) referred to our Department of Cardiology in the largest tertiary hospital in Slovenia for suspected CM between 2005 and 2020. Our tertiary centre receives approximately 75% of all referrals for suspected CM in the country.

All patients had TTE performed as part of the standard diagnostic workup. Echocardiographic characteristics of the cardiac mass were obtained, including mass location, surface (smooth or lobulated *vs*. villous) and appearance (homogenous *vs*. heterogenous). The mobility of the mass and the presence or absence of obstruction were also noted. Based on TTE findings, patients were diagnosed with either CM, other non-myxomal (NM) cardiac tumor, or cardiac masses of other etiology (thrombus, infective endocarditis, etc.). Diagnosis of CM was made individually by the cardiologist performing TTE based on typical morphological characteristics of the cardiac mass. In some cases, additional imaging methods were used, either due to poor TTE acoustic windows or atypical tumor presentation. TTE contrast imaging was not performed in any of the cases.

Patients with CM or NM cardiac tumors were referred for surgery and pathohistological samples of the tumors were collected and analyzed to determine the final diagnosis. The accuracy, sensitivity, and specificity of TTE were determined by comparing echocardiographic and pathohistological diagnosis. Furthermore, echocardiographic characteristics of pathohistologically proven CM were compared to NM cardiac tumors.

### Statistical analysis

Continuous variables are presented as mean ± standard deviation and categorical variables as numbers and percentage. The independent Student’s t-test was used to compare continuous variables. Categorical variables were analyzed using the χ^2^test. The sensitivity, specificity, negative predictive value, and positive predictive value of echocardiographic diagnosis of CM were calculated using the results of pathohistological examination as the gold standard. Accuracy was determined as the sum of true negative and positive tests divided by all tests. All statistical analyzes were performed using SPSS version 26.0 software. Values of p < 0.05 were considered statistically significant.

## Results

### Baseline characteristics

During the 15-year period, 73 patients were referred for evaluation of suspected CM. All patients underwent TTE and 63 (86%) were diagnosed with CM or NM cardiac tumor. Of the remaining 10 (14%) patients, five were diagnosed with thrombus and were treated accordingly with anticoagulation therapy. In three cases pseudotumor was diagnosed; one had a prominent Eustachian valve, one had a prominent Chiari network, and one had lipomatous hypertrophy of the interatrial septum. In two patients no obvious cardiac mass was found on repeat TTE.

Out of 63 patients diagnosed with either CM or NM, 35 (56%) underwent one or more additional imaging techniques to confirm the diagnosis, either due to suboptimal image quality on TTE or atypical tumor presentation. CMR was used most frequently (n = 23, 66%), followed by TEE (n = 20, 57%) and CT (n = 3, 9%). In two patients PET-CT was performed to detect possible distant metastases. No working diagnosis changed after additional imaging techniques.

After complete diagnostic workup, 53 (84%) of the 63 patients diagnosed with either CM or NM underwent surgery at our institution and were included for further analysis in our study. Of the 10 remaining patients, one underwent surgery at another institution, 4 had very small intracardiac masses, prompting periodic TTE follow-up, 4 were unfit for surgery, and 1 declined surgical intervention.

The mean age of the operated patients was 64 ± 14 years (26–85 years), 35 patients (66%) were female. The most common complaint was dyspnea (18 patients, 34%), followed by embolic events in 8 patients (15%), chest pain in 5 patients (9%), constitutional signs in 3 patients (6%) and palpitations in 2 patients (4%). Seventeen patients (32%) were asymptomatic. The mass was an incidental finding in 23 patients (43%), most commonly on TTE (61%) and chest CT (39%) performed for other indication.

### Echocardiographic characteristics

**FIGURE 1.**
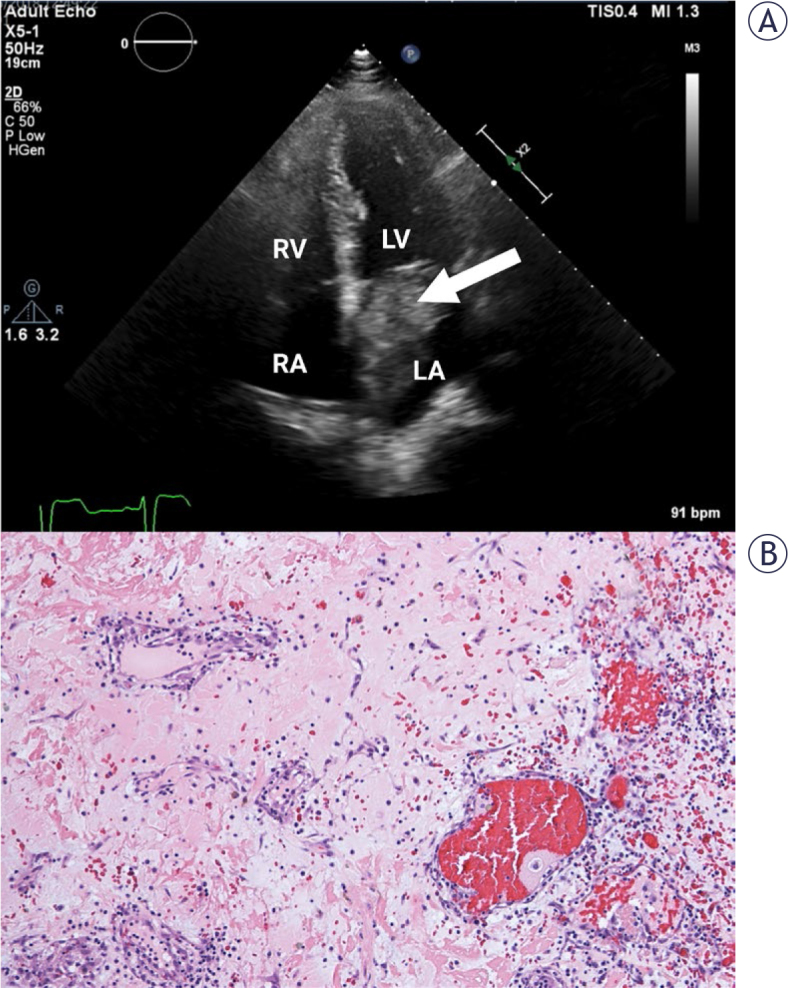
**(A)** Transthoracic echocardiography, apical 4-chamber view. Cardiac mass in left atrium is attached to the interatrial septum in the region of the fossa ovalis (arrow). Histopathological characterization confirmed cardiac myxoma. **(B)** Abundant myxoid stroma with clusters of myxoma cells forming cords and ring structures (HE 100×). LA = left atrium; LV = left ventricle; RA = right atrium; RV = right ventricle

Based on preoperative echocardiographic findings, the 53 operated patients were divided into two groups: a CM group (45 patients, 85%) and a NM group (8 patients, 15%). Preoperative echocardiographic characteristics are depicted in Table 1. All tumors were solitary. The mean tumor size in the CM group was 35.3 ± 18.6 mm (range: 10–81 mm). The majority of CM were located in the atria; 80% in the left atrium and 18% in the right atrium. One tumor was found in the left ventricle. Tumors found in the left atrium were most frequently attached to the atrial septum in the region of the fossa ovalis (n = 27, 75%) (Figure 1). Other attachment sites in the left atrium included the mitral valve (n = 4, two were attached to the posterior leaflet, one to the anterior leaflet, and one to the posterior annulus of the mitral valve), other areas of the atrial septum (n = 3, posterior part of the atrial septum), the free atrial wall (n = 1) and left atrial appendage (n = 1). The tumors were mostly mobile (n = 32, 71%). Mitral valve obstruction was observed in 10 (22%) patients and tricuspid valve obstruction in 1 (2%) patient.

**TABLE 1.** Preoperative echocardiographic characteristics of the cardiac myxoma (CM) and non-myxoma (NM) group

Echocardiographic characteristics	CM (n=45)	NM (n =8)
Mean size (mm)	35.3 ± 18.6 (range: 10–81)	30.3 ± 16.9 (range: 8–50)
Location		
Left atrium	36 (80)	2 (25)
Fossa ovalis	27 (75)	
Mitral valve	4 (11)	1 (50)
Posterior interatrial septum	3 (8)	
Free left atrial wall	1 (3)	1 (50)
Left atrial appendage	1 (3)	
Right atrium	8 (18)	4 (50)
Left ventricle	1 (2)	1 (12)
Aortic valve		1 (12)
Mobility		
Mobile	32 (71)	7 (88)
Non-mobile	5 (11)	
No data available	8 (18)	1 (12)
Surface		
Smooth or lobulated	28 (62)	1 (12)
Villous	8 (18)	3 (38)
No data available	9 (20)	4 (50)
Obstruction (present)	11 (24)	2 (25)
Mitral valve	10 (21)	1 (50)
Tricuspid valve	1 (9)	1 (50)

1 Values are presented as mean ± standard deviation or number (percentage).

The mean tumor size in the NM group was 30.3 ± 16.9 mm (range: 8–50 mm) (Table 1). All NM tumors were found in an atypical location, most frequently in the right atrium (n = 4, 50%). Two were attached to the left atrial free wall or posterior mitral valve leaflet and one to the aortic valve.

### TTE sensitivity, specificity, and accuracy analysis

Pathohistological samples were obtained from the resected tumors in all 53 surgical procedures performed at our institution. Pathohistological evaluation confirmed CM in 39 of 53 operated patients (73%) (Figure 1).

**FIGURE 2.**
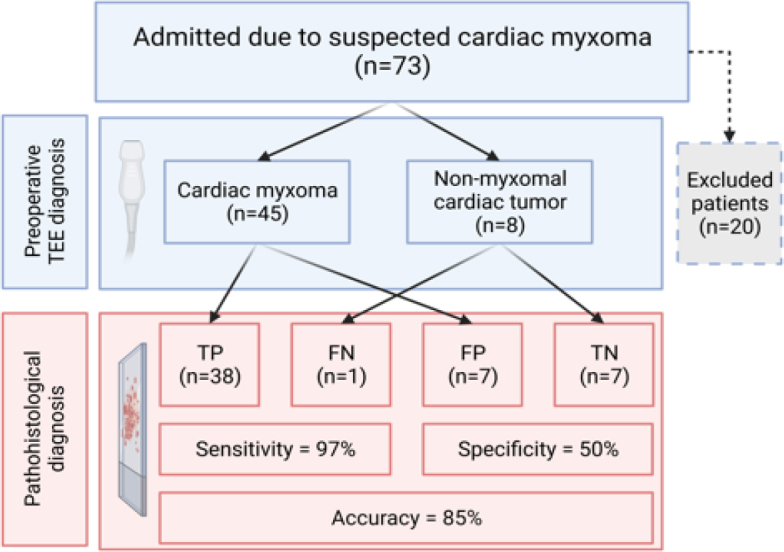
Flow diagram showing the diagnostic accuracy of preoperative TTE in patients with suspected CM. The predictive value and accuracy of preoperative TTE was calculated using the results of pathohistological examination as the gold FN = false negative; FP = false positive; TP = true positive; TN = true negative; TTE = transthoracic echocardiography

The calculated sensitivity and specificity of preoperative echocardiography in 53 patients who underwent surgery at our institution were 97% and 50%, respectively. The overall accuracy of TTE in diagnosing CM in those patients was 85% (Figure 2). In 7 patients (13%) diagnosed as CM on preoperative TTE, pathohistology revealed different NM cardiac tumors: papillary fibroelastoma in 5 cases, one case of angioleiomyoma and one malignant melanoma metastasis. Only one patient preoperatively classified as NM cardiac tumor had CM.

Pathohistologically confirmed NM cardiac tumors were significantly smaller than CM (24.3 ± 13.2 mm *vs*. 37.9 ± 18.3 mm, p = 0.017) (Table 2). There was also a statistically significant difference in tumor location between the two groups. All NM tumors were located at an atypical position (seven in the right atrium, five in the left atrium but at an atypical site and two in the left ventricle) and 72% of CM were found at the typical location within the left atrium (p < 0.001). The calculated sensitivity and specificity of tumor location in diagnosis of myxoma was 100% and 56%, respectively. There was no significant difference in other demographic (age, sex) or echocardiographic characteristics (mobility, surface, appearance) between groups.

**TABLE 2.** Comparison of demographic and echocardiographic characteristics between pathohistologically confirmed cardiac myxoma (CM) and non-myxoma (NM) groups

Characteristic	CM (n= 39)	NM (n = 14)	p value
Age (years)	63.1 ± 13.6	66.6 ± 15.1	0.434
Sex (female)	25 (64)	10 (71)	0.620
Location			
Typical	28 (72)	0	P < 0.001
Atypical	11 (28)	14 (100)	P < 0.001
Size (mm)	37.9 ± 18.3	24.3 ± 13.2	0.017

1 Values are presented as mean ± standard deviation or number (percentage).

## Discussion

Our single-center study confirms very good overall accuracy of TTE in CM diagnosis. This is clinically important as accurate assessment of cardiac masses is essential for appropriate clinical management and treatment of these patients.

Diagnosis of CM can be challenging since patients are frequently asymptomatic or have only non-specific signs and symptoms. Dyspnea, a frequent and non-specific symptom of cardiac disease, was the most common complaint in our CM group, which is consistent with previous reports.^9–11^ Clinical presentation itself rarely suggests the diagnosis of CM; therefore, cardiac imaging is essential in the evaluation of patients with suspected CM. Echocardiography is the most widely used imaging modality that provides important information about the location, size, and appearance of the cardiac mass, as well as possible complications (e.g. obstruction). Previous studies have shown that CM are typically solitary, located in the left atrium, smooth in surface and mobile.^12,13^ However, the morphological presentations of CM are often atypical and heterogeneous, leading to overlap with other NM cardiac tumors and cardiac masses.

The results of our study show very good overall accuracy (85%) of TTE in CM diagnosis with excellent sensitivity (97%). However, the specificity of TTE is modest (50%) and caution is warranted as misdiagnosis of CM is possible. In our study, 5 of the misdiagnosed cases of CM were actually papillary fibroelastoma, which is also a common primary benign cardiac tumor. One of the suspected CM was actually a metastasis of malignant melanoma, underlying the importance of surgical excision and pathohistological examination of all suspected CM.

According to our results tumor localization and tumor size are the best echocardiographic characteristics to distinguish between CM and NM cardiac tumors. CM are typically located in the left atrium attached to the interatrial septum at the region of fossa ovalis, which was also shown in our study.^14^ In our patients, 72% of CM were located typically. However, all tumors preoperatively misdiagnosed as CM were located in atypical locations, such as the right atrium and left ventricle. Tumors in the NM group were also significantly smaller compared to tumors in the CM group. However, there was no significant difference in age, sex, and other echocardiographic characteristics (mobility and surface) between the groups.

The differential diagnosis of CM is broad and definite diagnosis is crucial, as treatment varies depending on the diagnosis. Multimodality cardiac imaging improves the diagnostic accuracy of different cardiac masses. In the majority of our patients, at least one additional imaging modality was used as a part of the diagnostic workup. TEE improves image quality and provides more morphological information than TTE.^15^ Computed tomography and cardiac magnetic resonance provide additional information on topographic relationships and tissue characteristics, and may detect other pathological conditions within the thorax.^16,17^ Assessment of cardiac tumors by CMR is more accurate than echocardiography and can reliably distinguish between benign and malignant cardiac tumors.^18-20^

There are some limitations to this study. First, this is a retrospective study with a relatively small study population, precluding further analyses (e.g. Receiver Operating Characteristic). However, the population size is comparable to other studies on CM. Due to the low incidence of cardiac tumors, only multicenter studies can provide a larger scale patient population. Second, preoperative echocardiography was performed by different echocardiographers, potentially exposing the results to inter-investigator variability in determining the diagnosis. Due to the study inherently including participants already given a working diagnosis of CM, any cardiologist performing TTE was likely influenced by the information provided upon referral. A larger, multicenter, prospective study could serve to identify echocardiographic and clinical characteristics specific to CM, as well as other cardiac tumors, further increasing the utility of preoperative diagnostic modalities.

## Conclusions

TTE is very accurate in diagnosing CM. Tumor localization and size are the most important echocardiographic characteristics that can differentiate between CM and NM. The diagnosis of CM is less likely in atypical tumor location and smaller tumor size. In such cases, caution is advised and other non-invasive imaging modalities, such as CMR or CT, should be performed to confirm the diagnosis.
